# Supplementary material for: The impact of choosing words carefully: an online investigation into imaging reporting strategies and best practice care for low back pain
Source: PeerJ. 2017 Dec 6;5:e4151. doi: 10.7717/peerj.4151 (PMC5723139; doi:10.7717/peerj.4151)
Supplement: Supplemental Information 1 [file peerj-05-4151-s001.docx]

**Supplement 1.** **Descriptions and examples of the manipulations**

**A. Description of the manipulations**

*Pre-information:* Prior to receiving their own imaging results, patients were provided with age-relevant information about normal scan findings. This included explanation that many features identified on imaging occur naturally with age, are likely to have been present before the pain began, and are likely to still be there after the pain has resolved. Prevalence data for commonly reported findings was provided.

*Standard report:* Standard radiological reports were adapted from real reports from age-matched individuals.

*Epidemiological information:* A data summary table and short summary sentence providing information about the age-relevant prevalence of imaging characteristics in asymptomatic individuals was provided in addition to the standard report.

*Enhanced report:* The terminology used in the report summary was altered, with or without the addition of epidemiological information (as described). The altered summary used terms like “normal, age-related adaptations” rather than “degeneration” to describe imaging features likely to be age-appropriate.

*Best practice care:* The ‘patient’ was provided with information that aimed to set realistic expectations for recovery, reduce worries and concerns and guide return to usual activity. This information was developed to be consistent with best practice care, as recommended in current guidelines.

**B. Examples**

*Pre-information:*

**(For age range 18-25 years)**

Very often, scan reports describe findings that may sound abnormal, but actually occur naturally with age and are not unusual in people without any back injury or back pain.

For example, more than one third of 20 year olds WITHOUT any BACK PAIN show “disc degeneration” on their scans. “Disc bulges”, “disc protrusions” and “disc height loss” are almost as common (in people who are completely pain-free!)

When these scan findings are present in people with back pain – most of them would have been there before the pain began, and will still be there after the pain has gone.

*Standard report:*

**(For age range 18-25 years)**

**CT LUMBOSACRAL SPINE**

**Clinical:** Acute lower back pain for 2 weeks radiating to (R) buttock and posterior thigh.

**Technique:** Non-contrast CT scan. Slices obtained from L2 to S1.

**Findings:**

L2/3: Adequate sized canal with no disc herniation. The facet joints are well preserved.

L3/4: No abnormality. No disc herniation. The facet joints are well preserved.

L4/5: Mild annular laxity. Minor central disc bulging but this is not causing any compressive effects and there is no lateral extension to involve nerve roots. The facet joints are well preserved.

L5/S1: There is a pars defect on the right but the pars on the left side appears to be intact. There is a left sided paracentral disc protrusion, which is impinging on the left side of the thecal sac and possibly involving the exiting nerve root on the left side. No definite involvement of the right nerve root. The facet joints are well preserved.

**Comment:**

Unilateral pars defect at L5/S1 on the left. Minor disc bulging at L4/L5. Slightly more pronounced left sided paracentral disc protrusion on the left at L5/S1 with probable involvement of the exiting left nerve root. I note the patient’s symptoms are right sided.

*Epidemiological information:*

***(For age range 18-25 years)***

**CT LUMBOSACRAL SPINE**

**Clinical:** Acute lower back pain for 2 weeks radiating to (R) buttock and posterior thigh.

**Technique:** Non-contrast CT scan. Slices obtained from L2 to S1.

**Findings:**

L2/3: Adequate sized canal with no disc herniation. The facet joints are well preserved.

L3/4: No abnormality. No disc herniation. The facet joints are well preserved.

L4/5: Mild annular laxity. Minor central disc bulging but this is not causing any compressive effects and there is no lateral extension to involve nerve roots. The facet joints are well preserved.

L5/S1: There is a pars defect on the right but the pars on the left side appears to be intact. There is a left sided paracentral disc protrusion, which is impinging on the left side of the thecal sac and possibly involving the exiting nerve root on the left side. No definite involvement of the right nerve root. The facet joints are well preserved.

**Comment:**

Unilateral pars defect at L5/S1 on the left. Minor disc bulging at L4/L5. Slightly more pronounced left sided paracentral disc protrusion on the left at L5/S1 with probable involvement of the exiting left nerve root. I note the patient’s symptoms are right sided.

**ADDITIONAL INFORMATION:**

Radiologists must describe findings on spinal imaging in detail. However, many findings are found so commonly in adults WITHOUT any back problems, that whether or not these findings are related to a person’s pain requires a proper clinical assessment.

Imaging findings are very often NORMAL age-related changes. Here is a list of some common findings and how frequently they are found in 20 – 29 year olds *without* any back pain:


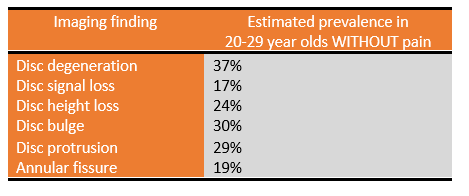


*Enhanced report:*

**CT LUMBOSACRAL SPINE**

**Clinical:** Acute lower back pain for 2 weeks radiating to (R) buttock and posterior thigh.

**Technique:** Non-contrast CT scan. Slices obtained from L2 to S1.

**Findings:**

L2/3: Adequate sized canal with no disc herniation. The facet joints are well preserved.

L3/4: No abnormality. No disc herniation. The facet joints are well preserved.

L4/5: Mild annular laxity. Minor central disc bulging but this is not causing any compressive effects and there is no lateral extension to involve nerve roots. The facet joints are well preserved.

L5/S1: There is a pars defect on the right but the pars on the left side appears to be intact. There is a left sided paracentral disc protrusion, which is impinging on the left side of the thecal sac and possibly involving the exiting nerve root on the left side. No definite involvement of the right nerve root. The facet joints are well preserved.

**Comment:**

Normal, age-appropriate, adaptations at multiple levels. The clinical history of an acute episode of low back pain with symptoms radiating to the (R) is noted. All reported findings may have been present prior to this painful episode.

**ADDITIONAL INFORMATION:**

Radiologists must describe findings on spinal imaging in detail. However, many findings are found so commonly in adults WITHOUT any back problems, that whether or not these findings are related to a person’s pain requires a proper clinical assessment.

Imaging findings are very often NORMAL age-related changes. Here is a list of some common findings and how frequently they are found in 20 – 29 year olds *without* any back pain:


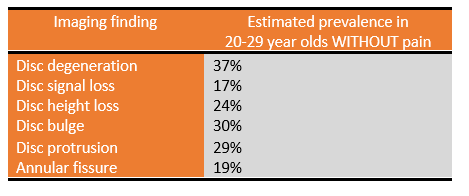


*Best practice care:*

**General Practitioner-delivered information**

You explain your back problem to your doctor who then examines your back. Your doctor understands your concern about how long it is taking for the pain to go away.

Your appointment then involves a thorough discussion of the following:

- Almost everyone experiences lower back pain at some stage – it really is quite normal and almost always gets better.
- While your back is painful and stopping you from doing your usual things - it is very unlikely to be a sign of serious damage or disease. It is common to have a lot of back pain even if there is not a bad injury. Pain is working in your favour to protect you.
- In general, back pain improves a lot during the first 2 weeks, but it sometimes takes several weeks longer to completely settle down. It can even come and go for a couple of months or more. It is usually possible however – and highly recommended - to gradually increase your activity during this time.
- Scans of your back (such as an x-ray, CT scan or MRI) are usually not helpful. They seldom show exactly where the problem is and even then, will not change your treatment plan. There are also some risks associated with having a scan that are best avoided.
- The best thing to do at this stage is to limit doing the things that make your pain worse, but gradually do more of other things. Getting moving is really important – try to do a little bit more every day.
- Respect your pain but don’t be afraid of it. Your pain protects you in the early stages after injury, but gradually returning to normal activity helps to coax this protective system back to normal mode too.

Your GP suggests that you make a further appointment in 6 weeks if you have not been able to return to all of your usual activities by this time.
