# Supplementary material for: The impact of choosing words carefully: an online investigation into imaging reporting strategies and best practice care for low back pain
Source: PeerJ. 2017 Dec 6;5:e4151. doi: 10.7717/peerj.4151 (PMC5723139; doi:10.7717/peerj.4151)
Supplement: Supplemental Information 2 [file peerj-05-4151-s002.docx]

**Supplement 2.** **Primary outcome – Back-Related Perceptions (BRP)**

Try to imagine that you are the person described in the scenario as you answer the following:

1. How would you rate the condition of your back?

| **Extremely poor**  **for my age** | |  |  |  |  |  |  | **Completely normal for my age** | |
| --- | --- | --- | --- | --- | --- | --- | --- | --- | --- |
| 1 | 2 | 3 | 4 | 5 | 6 | 7 | 8 | 9 | 10 |
|  |  |  |  |  |  |  |  |  |  |

2. How likely are you to try to return to normal work/activity/exercise as soon as you possibly can?

| **Not likely**  **at all** | |  |  |  |  |  |  | **Extremely**  **likely** | |
| --- | --- | --- | --- | --- | --- | --- | --- | --- | --- |
| 1 | 2 | 3 | 4 | 5 | 6 | 7 | 8 | 9 | 10 |
|  |  |  |  |  |  |  |  |  |  |

3. How worried are you that you will not be able to return to your normal work/activity/exercise?

| **Not at all**  **worried** | |  |  |  |  |  |  | **Extremely**  **worried** | |
| --- | --- | --- | --- | --- | --- | --- | --- | --- | --- |
| 1 | 2 | 3 | 4 | 5 | 6 | 7 | 8 | 9 | 10 |
|  |  |  |  |  |  |  |  |  |  |

**Primary outcome sum-score calculation:**

(Score Qu. 1) + (Score Qu. 2) + (11 – Score Qu. 3) = = BRP score
