# Supplementary material for: The impact of choosing words carefully: an online investigation into imaging reporting strategies and best practice care for low back pain
Source: PeerJ. 2017 Dec 6;5:e4151. doi: 10.7717/peerj.4151 (PMC5723139; doi:10.7717/peerj.4151)
Supplement: Supplemental Information 3 [file peerj-05-4151-s003.docx]

**Supplement 3.**  **Satisfaction question**

How satisfied are you with your doctor’s early management of your back pain?

| **Not satisfied**  **at all** | |  |  |  |  |  |  | **Very**  **satisfied** | |
| --- | --- | --- | --- | --- | --- | --- | --- | --- | --- |
| 1 | 2 | 3 | 4 | 5 | 6 | 7 | 8 | 9 | 10 |
|  |  |  |  |  |  |  |  |  |  |
