# Supplementary material for: The impact of choosing words carefully: an online investigation into imaging reporting strategies and best practice care for low back pain
Source: PeerJ. 2017 Dec 6;5:e4151. doi: 10.7717/peerj.4151 (PMC5723139; doi:10.7717/peerj.4151)
Supplement: Supplemental Information 5 [file peerj-05-4151-s005.docx]

**Supplement 5.** **(a) Demographic and baseline screening questionnaire**

**(b) Virtual patient scenario example**

**(c) Developed virtual patient scenario example**

**(a) Demographic and baseline screening questionnaire**

**What is your age?**

- 18-25
- 26-40
- 41-55
- 56-70
- 71 or older

**What is your gender?**

- Male
- Female
- Other

**In which country do you live?**

(Drop-down menu of all country names provided)

**Is English your first language?**

- Yes
- No

**Select the education level that best applies to you**

- I did not complete high school
- I completed high school
- I have enrolled in or completed a non-university qualification (e.g. TAFE certificate or diploma
- I have enrolled in or completed an undergraduate degree (e.g. Bachelor or Associates degree)
- I have enrolled in or completed a postgraduate degree (e.g. Grad. Dip., Masters, PhD)

**Have you ever had back pain?**

- Yes
- No

**Have you had back pain during the past 3 months?**

- Yes
- No

**Has pain been present on most days for the past 3 months?**

- Yes
- No

**Have you ever visited a doctor or other health-care provider about your back pain?**

- Yes
- No

**Have you ever had a scan (e.g. X-ray, CAT/CT scan or MRI) of your back?**

- Yes
- No

**When did you have the scan?**

- Less than 3 months ago
- 3-12 months ago
- Between 1 and 5 years ago
- More than 5 years ago

**(b) Virtual patient scenario example**

Imagine that you are a 21-year-old university student.

It is the mid-year break and you are moving into a unit with a friend.

As you lift a large box, you feel a sudden pain across your lower back. The sharp pain soon settles but you are left with a dull ache in your back, which spreads towards your right hip.

The next morning, all back movements cause severe pain in your lower back. By the end of the day, you have a constant ache spreading across your back and down into your right upper thigh. Bending, lifting and sitting are especially painful.

After 2 weeks your pain has improved but the ache in your back still comes and goes throughout the day. You haven’t been able to return to playing volleyball and you are really uncomfortable sitting in lectures due to increased pain in your back and upper leg.

**(c) Developed ‘virtual’ patient scenario example**

*Participants are randomly allocated to one of the following three ‘developed’ scenarios:*

1. Your doctor refers you for imaging of your lower back (as CT scan) and provides you with information about normal findings prior to receiving your scan
2. Your doctor refers you for imaging of your lower back (a CT scan)
3. You explain about your back problem to your doctor who then examines our back. Your doctor understands your concern about how long it is taking for the pain to go away. Your appointment then involves a thorough explanation of the following:

(See Appendix 4 for GP-delivered information).
